# A substantial proportion of apparently heterozygous *TP53* pathogenic variants detected with a next‐generation sequencing hereditary pan‐cancer panel are acquired somatically

**DOI:** 10.1002/humu.23910

**Published:** 2019-09-23

**Authors:** Bradford Coffee, Hannah C. Cox, Ryan Bernhisel, Susan Manley, Karla Bowles, Benjamin B. Roa, Debora Mancini‐DiNardo

**Affiliations:** ^1^ Myriad Genetic Laboratories, Inc. Salt Lake City Utah

**Keywords:** acquired somatically, hereditary pan‐cancer gene panel, Li–Fraumeni syndrome, next‐generation sequencing, pathogenic variant, *TP53*

## Abstract

Previous analysis of next‐generation sequencing (NGS) hereditary pan‐cancer panel testing demonstrated that approximately 40% of *TP53* pathogenic and likely pathogenic variants (PVs) detected have NGS allele frequencies between 10% and 30%, indicating that they likely are acquired somatically. These are seen more frequently in older adults, suggesting that most result from normal aging‐related clonal hematopoiesis. For this analysis, apparent heterozygous germline *TP53* PV carriers (NGS allele frequency 30–70%) were offered follow‐up testing to confirm variant origin. Ninety‐eight probands had samples submitted for follow‐up family member testing, fibroblast testing, or both. The apparent heterozygous germline *TP53* PV was not detected in 32.6% (15/46) of submitted fibroblast samples, indicating that it was acquired somatically, either through clonal hematopoiesis or via constitutional mosaicism. Notably, no individuals with confirmed germline or likely germline *TP53* PVs met classic Li–Fraumeni syndrome (LFS) criteria, only 41% met Chompret LFS criteria, and 59% met neither criteria, based upon provider‐reported personal and family cancer history. Comprehensive reporting of *TP53* PVs detected using NGS, combined with follow‐up analysis to confirm variant origin, is advised for clinical testing laboratories. These findings underscore the investment required to provide individuals and family members with clinically accurate genetic test results pertaining to their LFS risk.

## Graphical abstract

In next‐generation sequencing (NGS) hereditary cancer panel testing, *TP53* pathogenic variants whose NGS allele frequencies might appear consistent with those expected for germline variants, but the variant might be acquired somatically. Confirming *TP53* variant origin can markedly impact medical management for tested individuals and their family members. The report highlights the need for a conservative approach to initial reporting of *TP53*‐positive findings, along with investment in follow‐up testing to deliver clinically accurate results.

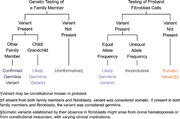

## 1 INTRODUCTION

Individuals who have Li–Fraumeni syndrome (LFS; MIM# 151623) have a high risk of developing sarcomas, brain tumors, adrenocortical carcinomas, premenopausal breast cancer, leukemia, and other cancers early in life (Sorrell, Espenschied, Culver, & Weitzel, 2013). Though no studies have directly measured life expectancy in LFS, it is known to be severely reduced (Evans & Ingham, 2013). Germline pathogenic variants (PVs) in *TP53* are the most common cause of LFS. Children who carry a germline PV in *TP53* have an approximately 42% risk of developing a *TP53*‐related cancer before age 16 (Le Bihan, Moutou, Brugières, Feunteun, & Bonaïti‐Pellié, 1995), and adult men have an approximately 73% lifetime risk of cancer (Chompret et al., 2000). Women who have germline *TP53* PVs have nearly a 100% chance of developing breast cancer during their lifetimes (Chompret et al., 2000). For these reasons, recent expert panel recommendations emphasize comprehensive, lifelong screening across numerous cancer types, beginning as soon as an individual has been confirmed to carry a *TP53* PV or meets the “classic clinical definition” of LFS with no *TP53* PV. This follows a modified version of the “Toronto protocol” (Kratz et al., 2017), which factors in the age and sex of the individual. In addition, National Comprehensive Cancer Network (NCCN) guidelines recommend that individuals with LFS receive aggressive cancer screening, including: annual breast MRI starting at age 20; colonoscopy every 2–5 years starting at age 25; whole‐body MRI; esophagogastroduodenoscopy; physical exam every 6–12 months; and a dermatologic exam starting at age 18 years (Daly et al., 2019). For women with LFS, risk‐reducing mastectomy also should be considered.

Due to the high cancer risks conferred by germline *TP53* PVs, hereditary pan‐cancer gene panels typically include *TP53*. Recent work showed that apparent germline *TP53* PVs were not uncommon among individuals who received hereditary cancer panel testing, accounting for 1% of germline PVs identified (Rosenthal, Bernhisel, Brown, Kidd, & Manley, 2017). Next‐generation sequencing (NGS) is used to accommodate the large number of genes often included in hereditary cancer panels; however, NGS is more sensitive than traditional sequencing methods and can detect variants at very low‐allele frequencies. Heterozygous germline variants will have NGS sequence reads with approximately a 1:1 ratio to those of the wild‐type allele, producing an NGS allele frequency of about 50%. Allele frequencies that differ significantly from 50% in blood‐derived samples suggest that the variant occurs in only a proportion of blood cells and therefore was acquired somatically. Somatic variants also can be detected in saliva‐derived samples, although the allele frequencies in saliva may be different than in blood, as saliva samples often contain a mix of epithelial and white blood cells.

A previous study showed that likely somatic PVs accounted for 0.71% of all PVs identified during hereditary cancer panel testing, with somatic PVs occurring most commonly in *TP53* and among older individuals (Coffee et al., 2017). This was consistent with previous work, which has shown that clonal expansion of blood cell subpopulations carrying somatic variants during hematopoiesis, also termed clonal hematopoiesis of indeterminate potential (CHIP), has been observed for a number of genes (Genovese et al., 2014; Jaiswal et al., 2014; Mitchell et al., 2018; Xie et al., 2014) and occurs with normal aging (Steensma et al., 2015). However, it was notable that in one case, the NGS allele frequency of a *TP53* PV increased from less than 30% (likely somatic range) to 45% (apparent germline range) when re‐tested after 3 months (Coffee et al., 2017). This led to the hypothesis that the NGS allele frequency range for somatically acquired *TP53* PVs may overlap with that expected for heterozygous germline variant alleles, thus potentially confounding test result interpretation.

Unlike germline PVs, variants acquired somatically through CHIP do not carry hereditary cancer risk, though they are associated with other risks such as hematologic cancer, cardiovascular disease, and all‐cause mortality (Genovese et al., 2014; Gillis et al., 2017; Jaiswal et al., 2014; Jaiswal et al., 2017; Takahashi et al., 2017). Given the severe clinical interventions for individuals with LFS, it is critical that apparent germline PVs in *TP53* be investigated further to determine whether they are truly in the germline. For this reason, our laboratory offers follow‐up testing to individuals with an apparent germline *TP53* PV identified during the course of clinical hereditary cancer testing, to determine whether the PV was germline or acquired somatically. This report presents the results of follow‐up testing.

## 2 MATERIALS AND METHODS

### 2.1 Editorial policies and ethical considerations

Only deidentified data collected as part of clinical testing were used in this analysis. All individuals provided informed consent for clinical testing. The analysis set did not include individuals from states with laws that prevent the use of deidentified genetic data. The corresponding and senior authors confirm that they had full access to all data used in the analysis and take responsibility for data integrity and the accuracy of the data analysis. All authors are employees of Myriad Genetic Laboratories, Inc., and receive salary and stock options as compensation.

### 2.2 Cohort and genetic testing

This analysis assessed individuals who had hereditary pan‐cancer panel testing (Myriad Genetic Laboratories, Inc., Salt Lake City, UT) as part of normal clinical care between September 2013 and February 2018. Testing was performed using blood or saliva samples. Validation of the NGS panel has been described previously and included analysis of *TP53* (Judkins et al., 2015). Sequence variants and large rearrangements (LRs) were analyzed relative to the wild‐type *TP53* gene sequence (RefSeq NM_000546.5). For sequence variants, apparent germline PVs were defined as those identified with an NGS allele frequency between 30% and 70% and which received a laboratory classification of Pathogenic or Likely Pathogenic. This threshold was assigned to minimize false negative results and was based on our laboratory's experience and expertise in clinical genetic testing. The broad range captures potential germline allele frequency imbalances that may occur due to technical or biological reasons. For LR variants, apparent germline PVs were identified through NGS dosage analysis, which used normalized read counts from sequencing amplicons to determine gene copy number. While this approach did not allow assignment of precise allele frequencies for LRs, dosage increases or decreases relative to those expected for a heterozygous germline deletion or duplication could be evaluated. A germline LR typically presents as a 50% increase or decrease in dosage.

All sequence PVs were confirmed by Sanger sequencing. Pathogenic LRs were detected by NGS and confirmed by microarray, multiplex ligation‐dependent probe amplification, and/or repeat NGS. Individuals who were found to have an apparent heterozygous germline PV in *TP53* were offered follow‐up testing at no additional cost to determine whether the variant was germline or somatic. Clinical information, including age, personal cancer history, and family cancer history, was obtained from provider‐completed test request forms and deidentified.

### 2.3 Follow‐up testing

**Figure 1:**
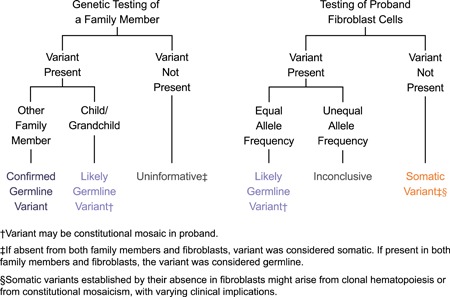
Follow‐up testing schema. The possible interpretations of follow‐up test results are provided for family testing and fibroblast testing in the proband

Follow‐up testing was performed either on blood or saliva samples from informative family members or on a cultured fibroblast sample from the proband (fibroblast cell culturing was performed by ARUP Laboratories, Salt Lake City, UT). DNA samples were extracted using standard methods. Follow‐up fibroblast and family testing consisted of Sanger single‐site analysis for the *TP53* PV that was initially identified in the proband. Figure 1 depicts the interpretation of the PV identified in the proband based on the results of follow‐up, confirmatory testing. When family member testing was performed, the *TP53* PV was “confirmed germline” if it also was identified in a relative who was not a child or grandchild. In this scenario, the PV was considered constitutionally present in all tissues, consistent with a molecular diagnosis of LFS. The PV was considered “likely germline” when it was present in a child or grandchild of the proband. In this scenario, it is likely that the PV is constitutional in the proband, yet it remains possible that it was acquired during early embryonic development and segregated to germ cells and other tissues, but not all tissues, rendering the proband constitutionally mosaic for the *TP53* PV. Family member testing was considered “uninformative” when the *TP53* PV was not detected in the tested family member.

When fibroblast testing was performed, the *TP53* PV was considered “somatic” if it was absent from the proband fibroblasts. This demonstrates that the PV is not present in all tissues; however, it remains undetermined whether the PV is restricted to blood (CHIP) or is present in some but not all tissues (constitutional mosaic). The PV was considered “likely germline” if it was present in the fibroblast sample from the proband. While identification of the PV in skin fibroblasts is strongly supportive of a constitutional variant, it does not definitively confirm the presence in the germline. Fibroblast testing results were “inconclusive” when initial NGS pan‐cancer panel testing showed an allele frequency consistent with a germline variant, but the ratio of wild‐type to PV allele in the fibroblast sample was significantly skewed from the expected 50:50 ratio. Such skewing might reflect the presence of more than one cell population in the skin biopsy, or it might result from technical or cell culture artifacts.

### 2.4 Analysis

Analyses were performed for all individuals with an apparent germline PV in *TP53*, were stratified by variant origin (confirmed germline, likely germline, somatic, uninformative, inconclusive, and unknown), and included age at testing and age at cancer diagnosis (if applicable). For the subset of individuals who had follow‐up testing, the results of follow‐up testing and NGS allele frequency were also assessed.

For all individuals who carried an apparent germline PV in *TP53*, clinical criteria for LFS were evaluated using classic LFS (Li et al., 1988) and revised Chompret LFS criteria (Bougeard et al., 2015). Classic LFS criteria were: proband diagnosed with sarcoma before 45 years of age, with a first‐degree relative diagnosed with any cancer before 45 years of age, and an additional first‐ or second‐degree relative diagnosed with any cancer before 45 years of age or a sarcoma at any age. Chompret LFS criteria were: (a) proband diagnosed with an LFS tumor (soft tissue sarcoma, osteosarcoma, brain tumor, adrenocortical carcinoma, leukemia, lung, or premenopausal breast [DCIS, invasive, or lobular invasive]) before 46 years of age, and at least one first‐ or second‐degree relative diagnosed with one LFS tumor before 56 years of age or with multiple primaries at any age; or (b) a proband with multiple tumors (except multiple breast tumors), the first of which occurred before age 46, and two of which belong to the LFS tumor spectrum; or (c) a proband with adrenocortical carcinoma, choroid plexus carcinoma, or rhabdomyosarcoma of embryonal anaplastic subtype, regardless of family history; or (d) breast cancer before 31 years of age.

## 3 RESULTS

**Figure 2:**
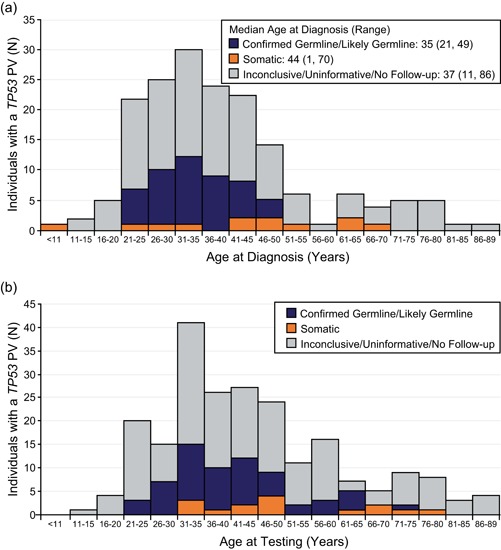
(a) Age at diagnosis and (b) age at testing for individuals with an apparent germline *TP53* PV identified by hereditary cancer genetic testing. Results are stratified according to the results of follow‐up testing

A total of 221 individuals were identified as carrying an apparent *TP53* PV (NGS allele frequency of 30–70%). The median age at testing was 41 years (range 15–88), and 28.5% (63/221) of individuals were older than 50 years (Figure 2). Overall, 79.6% (176/221) had a reported personal history of cancer at the time of testing. Of all individuals who had a reported personal history of cancer and age of diagnosis, 17.7% (29/174) were diagnosed after age 50. An additional 19.5% (43/221) of apparent germline *TP53* PV carriers had no reported personal cancer history, and two individuals had no information reported about personal cancer history.

### 3.1 Results of follow‐up testing

Ninety‐eight individuals had follow‐up testing to determine whether the *TP53* PV was present in the germline. This included family member testing only (52/98, 53.1%), fibroblast testing only (25/98, 25.5%), or both (21/98, 21.4%; Table 1). When follow‐up included family member testing only, the proband's PV was present in a parent, sibling or relative other than a child in 28.8% (15/52) of cases. The confirmed germline status for the PV in these individuals is consistent with a molecular diagnosis of LFS. The PV was identified in a child for an additional 25.0% (13/52) of cases, demonstrating that the variant was present in germ cells and was likely constitutional in the proband. Importantly, this result does not rule out the alternative possibility that the proband was constitutionally mosaic for *TP53*, and therefore these PVs were considered likely, and not confirmed, germline. However, the child of the proband would inherit the *TP53* PV in either scenario (germline PV or constitutional mosaic PV), which is consistent with a molecular diagnosis of LFS in the child. For 46.2% (24/52) of cases wherein the *TP53* PV was absent from tested relative(s), the results were considered uninformative. It is important to note that absence of the PV in both parents could represent acquisition of a de novo *TP53* PV in the proband, present in a proportion of a parent's germline cells (i.e., the parent is germline mosaic). It has been estimated that at least 14% of *TP53* PVs are de novo mutations (Renaux‐Petel et al., 2017).

**Table 1:** Summary of *TP53* follow‐up testing and interpretation

	Follow‐up test result interpretation				
Follow‐up testing performed	Confirmed germline	Likely germline	Somatic	Inconclusive/uninformative*	Total
Family testing only	15 (28.8%)	13 (25.0%)	0	24 (46.2%)	52
Fibroblast testing only	0	14 (56.0%)	9 (36.0%)	2 (8.0%)	25
Family and fibroblast testing	4 (19.0%)	10 (47.6%)	6 (28.6%)	1 (4.8%)	21
Total	19 (19.4%)	37 (37.8%)	15 (15.3%)	27 (27.6%)	98

* Fibroblast testing results were inconclusive when initial testing showed an allele frequency consistent with a germline variant, but the ratio of wild‐type to PV allele in the fibroblast sample was significantly skewed from the expected 50:50 ratio. Family member testing was considered uninformative when the *TP53* PV was not detected in the tested family member.

When follow‐up included fibroblast testing only, the *TP53* PV was detected in fibroblasts in 56.0% (14/25) of cases and was designated as likely germline (Table 1). The PV was absent from fibroblasts and thus designated as somatic in 36.0% (9/25) of cases. In 8.0% (2/25) of cases, the variant status was determined to be inconclusive; while the variant appeared to be germline in blood, the ratio was significantly skewed in fibroblasts, indicating possible mosaicism in skin tissue.

When follow‐up included both family member and fibroblast testing, the PV was absent in both fibroblasts and tested relatives in 28.6% (6/21) of cases and was considered somatic. In an additional 66.7% (14/21) of cases, the PV was present in both fibroblasts and tested relatives and was designated as confirmed germline or likely germline depending on the relationship of the tested relatives. For one case (4.8%; 1/21), the PV ratio was significantly skewed in fibroblasts and determined to be inconclusive.

### 3.2 Confirmed and likely germline *TP53* PV carriers

**Figure 3:**
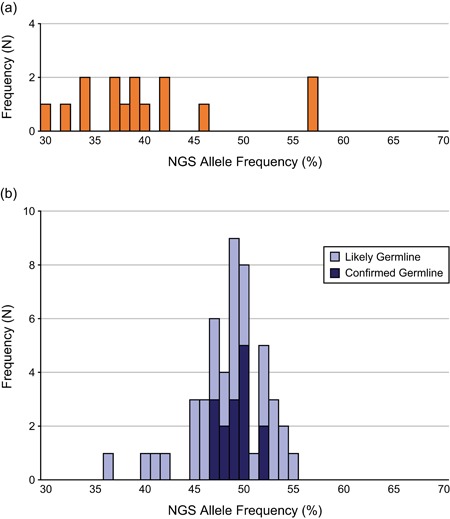
Allele frequencies for (a) somatic *TP53* PVs (*N* = 15) and (b) confirmed germline (*N* = 19) and likely germline (*N* = 37) *TP53* PVs. Only sequence variants are included. NGS, next‐generation sequencing; PV, pathogenic variant

Overall, follow‐up testing confirmed germline status for the original *TP53* PV identified during hereditary cancer testing in 19.4% (19/98) of individuals. In another 37.8% (37/98), the PV was considered likely germline (Table 1). The NGS allele frequencies for confirmed germline or likely germline sequence PVs in *TP53* (87.5%; 49/56) ranged from 36% to 55% at the time of initial testing, with most falling between 45% and 55% (Figure 3). Allele frequencies around 50% are consistent with those typically seen for heterozygous germline variants observed in other genes on the panel (data not shown). LRs such as single‐ or multi‐exon deletions or duplications comprised 12.5% (7/56) of confirmed germline or likely germline PVs. Follow‐up family member testing showed that 57.1% (4/7) of LRs represented a confirmed germline variant in the proband, and 42.9% (3/7) were likely germline.

Among the individuals who had a confirmed germline or likely germline PV in *TP53*, 78.6% (44/56) had a reported personal history of cancer. The median age at diagnosis among these individuals was 35 years (range 21–49 years), and 20.4% (9/44) were diagnosed after age 40. Based on personal and family cancer history information supplied by the provider on the test request form, 41.1% (23/56) of individuals with confirmed germline or likely germline *TP53* PVs met clinical criteria for Chompret LFS (Table 2). None met the criteria for classic LFS.

**Table 2:** Clinical Li–Fraumeni syndrome (LFS) classification, stratified by *TP53* follow‐up testing result

	Follow‐up test result interpretation			
	Confirmed germline/likely germline (*N* = 56)	Somatic (*N* = 15)	Inconclusive/uninformative (*N* = 27)	Unknown* (*N* = 123)
Did not meet clinical criteria for LFS	33 (58.9%)	12 (80.0%)	18 (66.7%)	86 (69.9%)
Met Chompret criteria for LFS	23 (41.1%)	3 (20.0%)	9 (33.3%)	37 (30.1%)
Met classic criteria for LFS	0	0	0	0

* Includes individuals for whom no follow‐up testing was performed.

### 3.3 Somatic *TP53* PV carriers

Among individuals with an apparent germline PV who had follow‐up fibroblast testing, *TP53* PVs were found to be somatic in 32.6% (15/46) of cases (Table 1). While confirmed germline/likely germline PVs shared similar NGS allele frequency ranges, grouping around 50%, the somatic PV allele frequencies were dispersed more evenly (Figure 3).

Eighty percent (12/15) of individuals with a somatic *TP53* PV had a reported personal history of cancer, with a median age at diagnosis of 44 years (Figure 2). For six somatic PV carriers, early‐onset breast cancer (ages 25, 28, 32, 33, 41, and 42) was reported. An additional six somatic PV carriers were not diagnosed with cancer until after age 45. There were no clear differences in the NGS allele frequencies of somatic PVs based on age at diagnosis. Allele frequencies for somatic PVs found in individuals diagnosed at an early age ranged from 30% to 46% compared with 34–57% for those diagnosed at a later age. The remaining three individuals with somatic PVs and no reported personal cancer history were in their late 40s at the time of testing (allele frequencies 37%, 40%, and 57%). No somatic *TP53* PV carriers met clinical criteria for classic LFS; however, three individuals (20.0%) met criteria for Chompret LFS (Table 2).

## 4 DISCUSSION

This report describes the results of follow‐up testing to determine variant origin for individuals found to harbor apparent germline *TP53* PVs as part of NGS hereditary cancer genetic testing. Follow‐up testing consisted of family testing and testing of fibroblasts from the proband. Family member testing can confirm that a PV is present in the germline (through the presence in the proband's sibling or parent) or likely germline (through the presence in the proband's child or grandchild). The result is uninformative when a parent appears to be negative for the PV detected in the proband. This is because three possible explanations exist. First, the PV might have been acquired during gametogenesis in the parent and is present only in a proportion of the parent's germ cells (i.e., parent is germline mosaic). It has been estimated that at least 14% of *TP53* PVs represent de novo mutations (Renaux‐Petel et al., 2017). Second, the PV might have been acquired early in development and then segregated into some, but not all tissues (i.e., proband is constitutional mosaic). Third, the PV might have been acquired somatically in the proband's blood later in life, via CHIP.

On the other hand, fibroblast testing can determine that a PV is likely germline (present in fibroblasts) or that the PV was acquired somatically (absent in fibroblasts). Fibroblast testing, with or without additional family testing, showed that a substantial proportion (32.6%) of apparent germline *TP53* PVs were acquired somatically. Although this proportion might reflect some self‐selection among individuals who elected to undergo follow‐up testing, it underscores the important fact that NGS allele frequency alone cannot determine the germline or somatic status of a *TP53* PV. Therefore, while follow‐up fibroblast testing cannot definitively distinguish between CHIP and constitutional mosaicism to establish the origin of the *TP53* PV, it can provide additional information that, when considered in context of clinical factors such as age and cancer history, informs screening and management approaches.

Most individuals who carried a somatic PV in *TP53* reported a personal history of cancer. Furthermore, half of the somatic PV carriers were diagnosed when younger than 45 years of age. While only three individuals with a somatic PV met Chompret criteria for LFS, cancer at younger ages is consistent with the expected phenotype for LFS and meets NCCN criteria for *TP53* testing (Daly et al., 2019). Overall, these data indicate that both genetic test results and the clinical presentation for individuals who carry somatically acquired *TP53* PVs can resemble those of LFS or other hereditary cancer syndromes.

Similar to the somatic PV carriers, the majority of individuals with a confirmed germline PV in *TP53* also reported a personal history of cancer. This included several individuals who were diagnosed after age 40, which is inconsistent with the expected clinical presentation of individuals with LFS. Overall, 58.9% (33/56) of confirmed germline or likely germline *TP53* PV carriers did not meet current clinical criteria for LFS. Conflicting genetic findings and clinical phenotype may result in uncertainty regarding medical management. However, the findings presented here demonstrate that a large proportion of confirmed germline or likely germline PVs were identified among individuals whose reported personal and family cancer history on test requisition forms suggest that they do not meet the traditional criteria for LFS. Unlike previous studies that also suggest that the LFS phenotype is broader than is currently defined (Amadou, Waddington Achatz, & Hainaut, 2018; de Andrade et al., 2019, 2017; Rana et al., 2018), this analysis is not confounded by the presence of somatic carriers of *TP53* PVs.

There is significant interest in understanding the origin of somatically acquired PVs in *TP53*. Individuals with somatic *TP53* PVs who were diagnosed with cancer at young ages might be constitutionally mosaic for the PV. For the four women described here who were diagnosed with breast cancer in their late 20s and early 30s, detection of the PV in the relevant tissue type would support its contribution to the disease. The absence of *TP53* PVs from the fibroblasts of three individuals in their late 40s with no personal cancer history could be consistent either with constitutional mosaicism or with CHIP. *TP53* is one of the most common genes known to accrue somatic PVs in blood due to CHIP (Genovese et al., 2014; Jaiswal et al., 2014; Xie et al., 2014). CHIP has been shown to be present in about 10% of individuals older than 65 years, and its prevalence increases with age (Genovese et al., 2014; Jaiswal et al., 2014; Xie et al., 2014). Association of somatically acquired, low‐allele‐frequency (10–30%) *TP53* PVs with aging has been seen previously in individuals tested using NGS hereditary cancer gene panels (Coffee et al., 2017). It follows that further expansion of a hematopoietic cell subclone carrying a *TP53* PV could occur over time, reaching an NGS allele frequency that overlaps with those seen for heterozygous germline variants. The observation that subclones with *TP53* PVs in mouse bone marrow chimeras expand over time, specifically after exposure to chemotherapy, further supports this hypothesis (Wong et al., 2015).

Although it is likely that CHIP underlies the acquisition of somatic *TP53* PVs in this testing population, particularly among older individuals, it remains possible that some individuals are constitutionally mosaic for the variant. It is important to distinguish between these two mechanisms in tested individuals, as the implications for clinical management likely will differ. Constitutional mosaicism, wherein the *TP53* PV is present in tissues vulnerable to LFS‐associated cancers, might be expected to cause early‐onset cancers. CHIP, as a normal byproduct of aging, might hold other implications for medical management. While the possible origins of somatic variants are not easily distinguishable, confirming whether a PV in *TP53* within the 30–70% AF range is somatic versus germline can inform whether the extensive clinical interventions for germline PV carriers are appropriate. This distinction can both prevent unnecessary interventions for individuals who have nongermline PVs, as well as under‐treatment for individuals who harbor germline *TP53* PVs but do not meet clinical LFS criteria.

A primary strength of the analysis is the large clinical data set that afforded the broad analysis and follow‐up testing in individuals tested for hereditary cancer risk. Personal and family cancer history entered on the test request form by the ordering provider further enhances data analysis and interpretation but comes with a limitation: the possibility that the information is less than complete. For some tested individuals, the family cancer history might be more extensive than was reported and could bring the family cancer history phenotype in closer alignment with LFS diagnostic criteria. Cytotoxic cancer therapy is known to be associated with CHIP (Coombs et al., 2017; Gillis et al., 2017; Slavin et al., 2019; Takahashi et al., 2017) and should be considered by ordering providers as a potential contributor to acquisition of somatic *TP53* PVs. Unfortunately, cancer treatment information was not available in this clinical testing data set; this is an important area for investigation using study cohorts that are designed to capture such information.

In summary, this study demonstrates that *TP53* PVs with NGS allele frequencies consistent with those expected for a germline PV can be either inherited or acquired somatically. Therefore, clinical reporting of *TP53* PVs identified by NGS should include the possibility of either germline or somatic origin and the corresponding clinical implications. For individuals who carry germline *TP53* PVs, confirmation of the test result is essential to providing appropriate medical management. Determining that a *TP53* PV was acquired somatically may help prevent unnecessary medical intervention for the proband and undue anxiety for family members who otherwise may think that they are at risk of carrying the PV. These findings highlight the need for a conservative approach to the initial reporting of *TP53*‐positive NGS test results, combined with investment in follow‐up testing to deliver clinically accurate results.

## CONFLICT OF INTERESTS

All authors are employed by Myriad Genetic Laboratories, Inc. and receive salaries as compensation.
